# Different Size Formulations of Fluopyram: Preparation, Antifungal Activity, and Accumulation in the Fungal Pathogen *Botrytis cinerea*

**DOI:** 10.3390/molecules28166099

**Published:** 2023-08-17

**Authors:** Yinmin Wang, Sida Zhang, Yong Xu, Haiyun Li, Ruihua Zhang, Dong Chen, Jianfu Xu, Xuemin Wu

**Affiliations:** 1 Innovation Center of Pesticide Research, Department of Applied Chemistry, College of Science, China Agricultural University, Beijing 100193, China; yinminwang@cau.edu.cn (Y.W.);; 2 Key Laboratory of National Forestry and Grassland Administration on Pest Chemical Control, China Agricultural University, Beijing 100193, China; 3 State Key Laboratory of NBC Protection for Civilian, Beijing 102205, China; stars5123@163.com (S.Z.);

**Keywords:** *Botrytis cinerea*, gray mold, pesticides, nanoparticles, fluopyram

## Abstract

Nanotechnology is revolutionizing the efficient production and sustainable development of modern agriculture. Understanding the pesticide activity of both nano- and conventional methods is useful for developing new pesticide formulations. In this study, three solid fluopyram formulations with varying particle sizes were developed, and the mechanisms underlying the difference in the antifungal activity among these formulations were investigated. Wet media milling combined with freeze drying was used to prepare fluopyram nanoparticles (FLU-NS) and a micron-sized solid formulation (FLU-MS), and a jet grinding mill was employed to fabricate fluopyram wettable powder (FLU-WP). The mean particle sizes of FLU-NS, FLU-MS, and FLU-WP were 366.8 nm, 2.99 μm, and 10.16 μm, respectively. Notably, FLU-NS displayed a toxicity index against *Botrytis cinerea* (gray mold) that was approximately double those of FLU-MS and FLU-WP. Similar trends were noticed in the antifungal tests on *Alternaria solani*. The uptake of FLU-NS by *B. cinerea* was approximately twice that of FLU-MS and FLU-WP, indicating that fluopyram nanoparticles are more easily taken up by the pathogen (*B. cinerea)*, and display better bioactivity than the larger fluopyram particles. Therefore, the nanosizing of pesticides appears to be a viable strategy to enhance efficiency without increasing the amount of pesticide used.

## 1. Introduction

Worldwide, an annual economic loss of 200 billion USD in grain production is attributed to plant diseases, insect infestations, and weed growth, with fungi responsible for approximately 70% of all crop diseases [1]. The use of pesticides is an effective tool for managing plant diseases and pests. However, the low dispersibility and bioavailability of traditional pesticides have led to a utilization rate of less than 30% for crops [2]. Moreover, less than 0.1% of the active ingredients in pesticides reach the targeted site. Drugs that target the inside of cells, such as succinate dehydrogenase (SDH), need to cross both the fungal cell wall and the plasma membrane to reach their destination (such as the mitochondria, in the case of SDH). Unfortunately, the large particle sizes of traditional pesticide formulations impede their ability to cross fungal cell walls, thereby reducing their efficacy. In recent years, rapid progress in the field of nanotechnology has led to an increased interest in nanopesticides [3,4,5], as the smaller particle sizes allow for easier target access and higher efficacy than those of traditional pesticides. These nano-based properties have been proposed as potential solutions to the issues associated with conventional pesticide formulations.

Essentially, two approaches can be used to synthesize nano-based formulations: top-down methods and bottom-up processes. Top-down methods primarily include media milling, high-pressure homogenization, and sonication, while bottom-up processes involve melt dispersion, solvent displacement, complex coacervation, interfacial polymerization, and emulsion diffusion [6,7]. Wet media milling is an effective technique for the nanonization and enhancement of drug bioavailability with poor water solubility [8,9,10]. The suspension obtained via milling is inherently unstable because of the high surface free energy of the nanoparticles [11], which can lead to aggregation or Ostwald ripening. A typical approach to preventing these phenomena includes the addition of surfactants with charged head groups to the nanoparticle suspensions [12,13,14]. Surfactants attach to drug surfaces and inhibit their subsequent aggregation through steric hindrance or the electrostatic modification of the surface [15,16,17].

*Botrytis cinerea* (gray mold), a necrotrophic fungal pathogen, is one of the most dangerous plant pathogens worldwide, owing to its high pathogenicity [18]. It infects more than 200 plant species, including those used for commercial crops, such as tomatoes, berries, and petunia flowers, resulting in significant economic losses [19,20,21].

**Figure 1:**
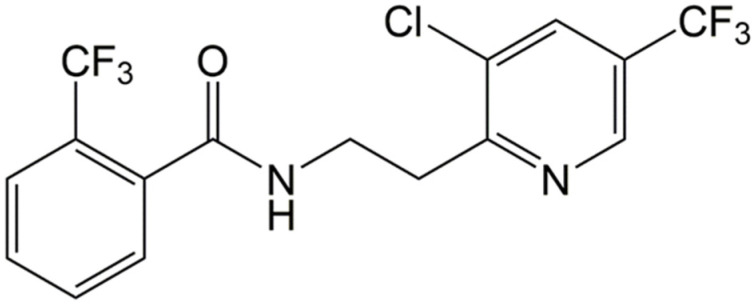
The chemical structure of fluopyram.

Complex II of the mitochondrial respiratory chain, also known as SDH or succinate-ubiquinone oxidoreductase (SQR), is located in the inner membrane of the mitochondria in eukaryotic cells [22,23]. Complex II plays a crucial role in the two major metabolic pathways which generate ATP, including the tricarboxylic acid cycle (TCA) and oxidative phosphorylation (OXPHOS) [24]. Fluopyram (FLU), a pyridinylethylbenzamide (Figure 1), is a newly developed broad-spectrum fungicide, which targets Complex II. Fluopyram was selected as the model pesticide for nanoformulation in this study because of its bioactivity against all growth stages of fungi and several pathogenic species belonging to ascomycetes and deuteromycetes, such as *Botrytis* spp., *Sclerotinia* spp., and *Monilinia* spp., in vegetables, melons, and stone fruit crops.

Research on the interactions between plants and their pathogens has shown that the fungi that cause plant diseases exhibit biofilm-like characteristics in their growth, such as robust hyphal networks and varied fungal layers [25]. However, fluopyram targets the inner mitochondrial membrane in eukaryotic cells, penetrates the fungal cell wall and cell membrane, and reaches the inner mitochondrial membrane. Additionally, the small size of nanoparticles improves their ability to permeate cell membranes, giving them easier and quicker access to their target sites [26,27]. Therefore, it is important to study the fungicidal effects of fluopyram nanoparticles and conventional particles for the efficient application of pesticides with intracellular targets.

In this study, we prepared fluopyram nano-(FLU-NS) and micro-(FLU-MS) solid formulations by integrating wet media milling and freeze drying. For comparison, fluopyram wettable powder (FLU-WP) was prepared using a jet grinding mill. This study investigated the influence of different particle sizes on the antifungal activity of fluopyram formulations. The particle sizes and morphologies of the three formulations were characterized using dynamic light scattering (DLS) and scanning electron microscopy (SEM). Further, the antifungal activities of the three formulations against the fungal pathogens *B. cinerea* and *Alternaria solani* were evaluated using a potato dextrose agar (PDA) assay. Lastly, fluopyram uptake tests were performed to measure the intracellular accumulation of the formulations.

## 2. Results and Discussion

### 2.1. Preparation of Fluopyram Solid Formulation

The influence of the different surfactants on the nanosuspensions was evaluated via assessment of the average particle size and PDI (fluopyram to surfactant ratio of 10:1). We noticed that the particle size of the suspension varied significantly with the surfactant used (Table 1), with D425 and F127 exhibiting superior dispersion effects in all the surfactants. Through the use of the surfactants D425 and F127, the suspension’s average particle size was reduced to below 600 nm, and its particle size distribution (PDI) became narrow, with a value of less than 0.25.

Subsequently, D425 was chosen as the surfactant for the formulation of the suspension, and its amount was optimized. The mean particle size of the suspension obtained at a fluopyram to D425 ratio of 7.5:1 was 576 nm. Notably, a uniform and stable suspension was not obtained at a ratio of 20:1. Further, the particle size of the suspension decreased as the quantity of the added surfactant reduced, making the suspension less stable because the low concentration of surfactant was unable to reach adsorption saturation on the particle surface, leading to particle aggregation and precipitation.

**Figure 2:**
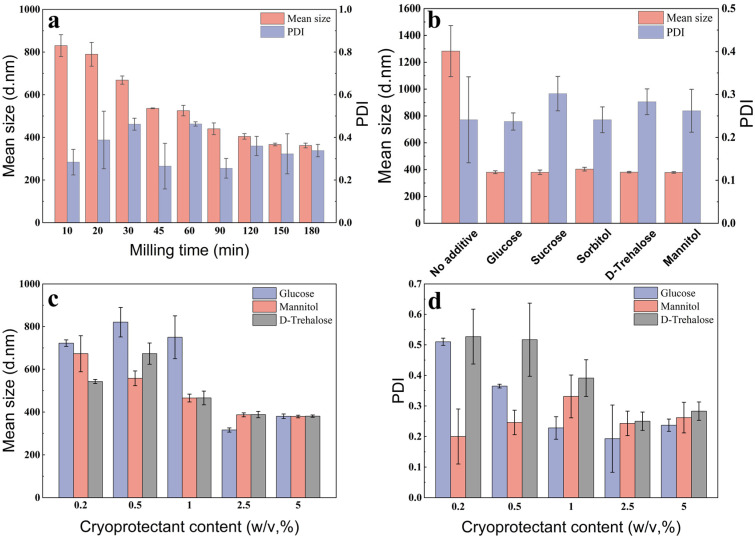
The effect of the milling time and cryoprotectant on the characteristics of the nanosuspension: (**a**) milling time, (**b**) category of cryoprotectant, (**c**) content of cryoprotectant, and (**d**) PDI.

The particle size in the suspension decreased as the grinding time increased, achieving a minimum value at 150 min (Figure 2a), with a mean particle size of 366 nm. However, the stress generated through freeze-drying can cause the NPs to aggregate into large particles, making it difficult to redisperse them in aqueous media [28,29]. Many studies have shown that cryoprotectants can effectively protect NPs and enable sufficient recovery in water [30,31]. Therefore, five cryoprotectants were added, to maintain the suspension performance. As illustrated in Figure 2b, all cryoprotectants provided excellent protection to the solid formulations during the freeze-drying process. The mean particle size of the redispersed suspension without a cryoprotectant was three times larger than that of the suspension prior to freeze drying. Moreover, the particle sizes of the solid formulations with added cryoprotectants were not significantly bigger. The cryoprotectants glucose, D-trehalose, and mannitol showed a superior freeze-dried protective efficacy, with a mean particle size of 380.5, 380.9, and 379.2 nm, and a PDI of 0.237, 0.283, and 0.262, respectively. Thus, these cryoprotectants were selected for further optimization.

The mean particle size and PDI of the suspension decreased with the increasing incorporation of the three cryoprotectants, which effectively prevented nanoparticle agglomeration at a content surpassing 2.5% (Figure 2c,d). The formulation with added glucose had the smallest particle size but was accompanied by severe caking and poor flowability. Thus, 2.5% D-Trehalose was selected as the cryoprotectant.

To prepare the fluopyram micron formulation, HPMC was selected as the surfactant, with 7.5:1 as the ratio of FLU to surfactant, which was consistent with the ratio used with FLU-NS (Table 2).

The effect of the rotating speed on the particle size in the suspension during ball milling showed that the particle size of the fluopyram in the suspension gradually decreased with the increasing rotation speed of the ball mill (Table 3). To obtain FLU-MS that significantly differed from FLU-NS in terms of the particle size, a micron suspension was prepared at 300 rpm, followed by the addition of D-Trehalose for freeze drying to obtain FLU-MS.

To gain further insights into the differences between the new and traditional formulations, FLU-WP was prepared via jet milling, with a composition of 20% fluopyram, 3% dispersant NNO, 3% wetting agent EFW, 10% white carbon, and diatomite supplement.

### 2.2. The Particle Size and ζ Potential

**Figure 3:**
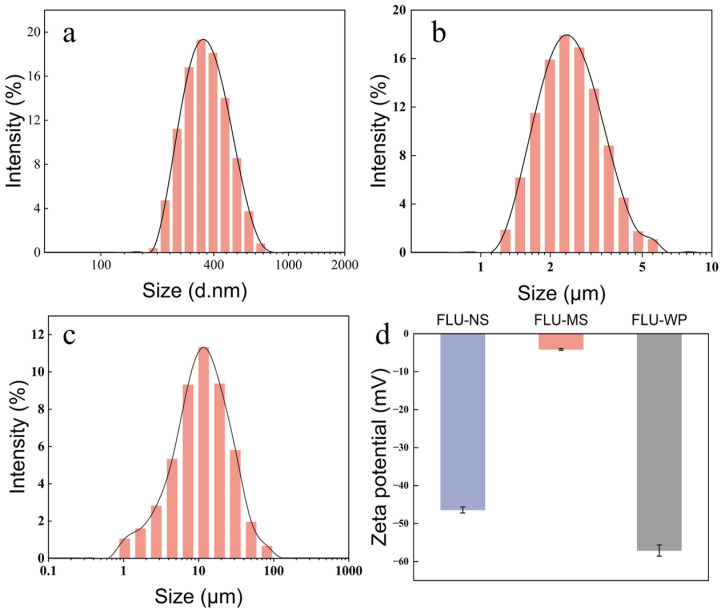
Diagrams showing the particle size distribution and zeta potential of three solid formulations: (**a**) FLU-NS, (**b**) FLU-MS, (**c**) FLU-WP, and (**d**) ζ potential.

The mean particle size and PDI of the nano- and microsolid formulations diluted in water, measured via DLS, were 366.8 nm and 2991 nm, respectively, with PDI values of 0.323 and 0.268 (Figure 3a,b). The D50 of the wettable powder diluted in an aqueous solution, as measured using a laser particle size analyzer, was 10.16 μm (Figure 3c). Further, the ζ potentials of the aqueous diluted solid formulation for FLU-NS, FLU-MS, and FLU-WP were −46.4 mV, −4.2 mV, and −57.1 mV, respectively (Figure 3d). 

The high negative ζ potential values of FLU-NS and FLU-WP can be attributed to the anionic surfactant, which provides a strong electrostatic repulsion, thereby stabilizing the fluopyram particles. FLU-MS, however, exhibited a lower ζ potential value, likely due to the non-ionic surfactant HPMC, which does not generate ions in aqueous solution. Compared to electrostatic repulsion, HPMC relies on effective steric hindrance to stabilize the particles.

### 2.3. Morphology

**Figure 4:**
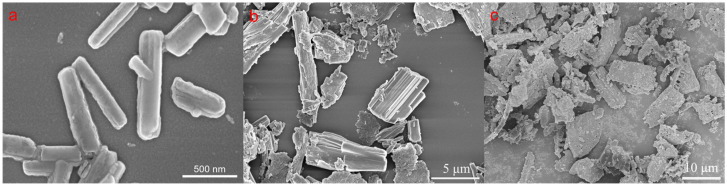
The morphologies of the three solid formulations characterized via electron microscopy: (**a**) FLU-NS, (**b**) FLU-MS, and (**c**) FLU-WP.

Scanning electron microscopy revealed the morphologies of the three fluopyram solid formulations. The FLU-NS particles exhibited a regular rod-like shape, smooth surface, uniform size, and monodispersity, as shown in Figure 4a. In contrast, the FLU-MS and FLU-WP particles had rough and uneven surfaces. The presence of a large amount of diatomaceous earth made it challenging to observe the morphology of FLU-WP, as shown in Figure 4c.

### 2.4. Crystalline State

**Figure 5:**
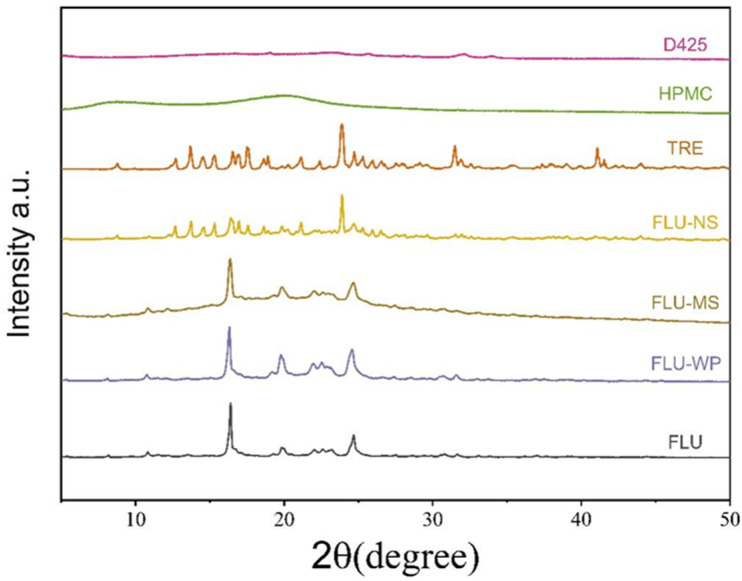
Powder X-ray diffraction patterns of three FLU formulations. Powders were loaded into a Kapton capillary.

The X-ray diffraction spectra of fluopyram, FLU-WP, FLU-MS, and FLU-NS were obtained and analyzed (Figure 5). The characteristic diffraction peaks of fluopyram were identified at 16.38°, 19.82°, 21.98°, and 24.62°. The diffraction peaks of FLU-WP and FLU-MS are consistent with those of fluopyram. The strongest diffraction peak at 23.9° for FLU-NS was primarily attributed to D-Trehalose, which is consistent with what is reported in the research [32]. Meanwhile, the characteristic diffraction peaks of fluopyram were also clearly observed, at 16.38°, 19.82°, and 21.98°. D425 and HPMC did not show any characteristic diffraction peaks; thus, they did not interfere with the characteristic diffraction peak of fluopyram in the formulations.

The zirconium oxide media rotates at a rapid rate during grinding, causing significant heat and mechanical shearing forces. This process can lead to increased lattice vibrations and a change from the crystalline state to an amorphous state [33,34]. The physical structure of organic components may undergo modifications, such as crystal defects or crystalline transitions, as a result of wet media milling. X-ray diffraction analysis revealed that the fluopyram in the three solid formulations primarily retained a crystalline state, without transformation to the crystal form.

### 2.5. Stability of Three Solid Formulations

**Figure 6:**
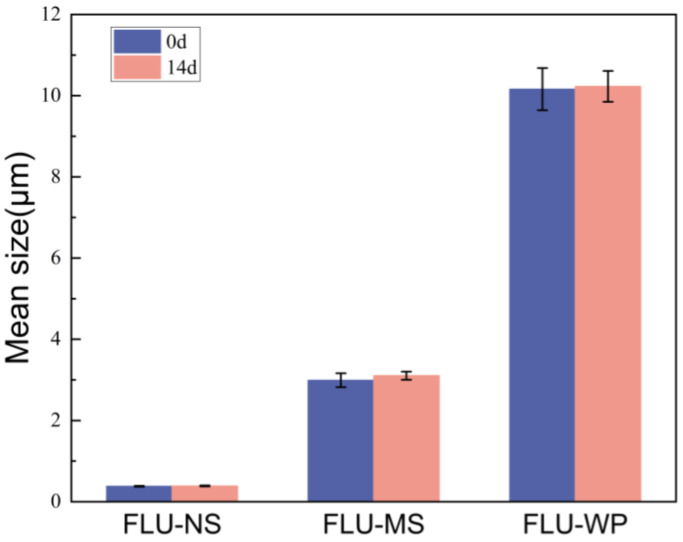
The thermal storage stability (54 °C) of the three FLU formulations.

According to the national standard of National Standards of the People’s Republic of China, GB/T 19136-2021 “Testing method of the accelerated storage stability at elevated temperature for pesticides”, we experimented to determine the thermal storage stability of three solid fluopyram formulations. The formulations were stored at 54 °C for 14 days, and we recorded the changes in their mean particle size and distribution. The results indicate that the mean particle sizes in the formulations did not significantly increase during storage (Figure 6). This suggests that the formulations possess an excellent storage stability and may effectively prevent the particle aggregation caused by Oswald ripening [35].

### 2.6. Antifungal Activity Evaluation

**Figure 7:**
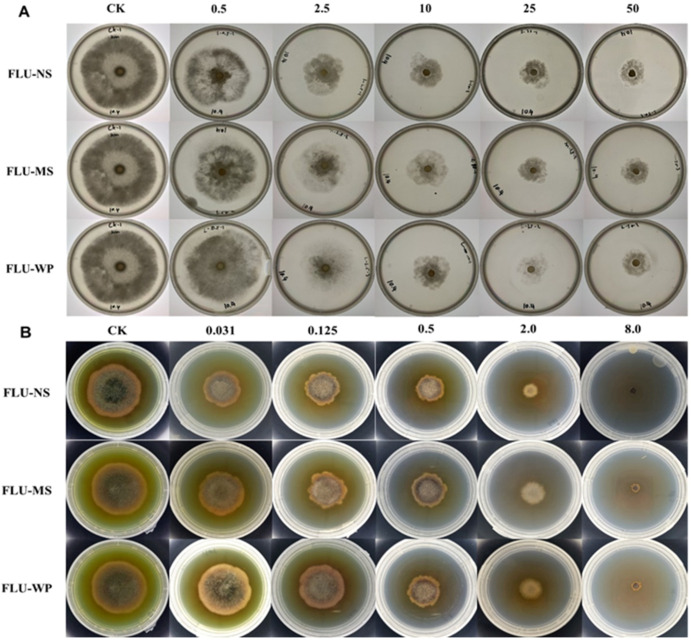
The antifungal activity of three fluopyram solid formulations against *Botrytis cinerea* (**A**) and *Alternaria solani* (**B**). The mycelium growth inhibition method was used to test the FLU activity and determine the EC_50_ calculated using IBM SPSS Statistics Version 26.0.

The antifungal activities of the three solid formulations of fluopyram against *B. cinerea* and *A. solani* were examined using a PDA assay with EC_50_ values (Table 4). FLU-NS exhibited the highest antifungal activity against *B. cinerea* and *A. solani*, with an EC_50_ value of 5.389 and 0.244 μg/mL, respectively. The antifungal activity indexes of FLU-NS and FLU-MS compared to FLU-WP were 1.97-fold and 1.07-fold against *B. cinerea*, and they were 3.42-fold and 1.37-fold against *A. solani*, respectively (Figure 7).

The results of the antifungal assay confirmed that a decrease in the mean size of the fluopyram particles from 10 μm to ~300 nm increased the antifungal activity 1.97–3.42-fold. However, reducing the particle size to 3 μm had a negligible effect on the antifungal activity. These findings suggest that the antifungal activity of fluopyram is significantly improved when its particle size is reduced to the nanoscale, compared to the microscale.

### 2.7. Different-Size-FLU Uptake by B. cinerea

**Figure 8:**
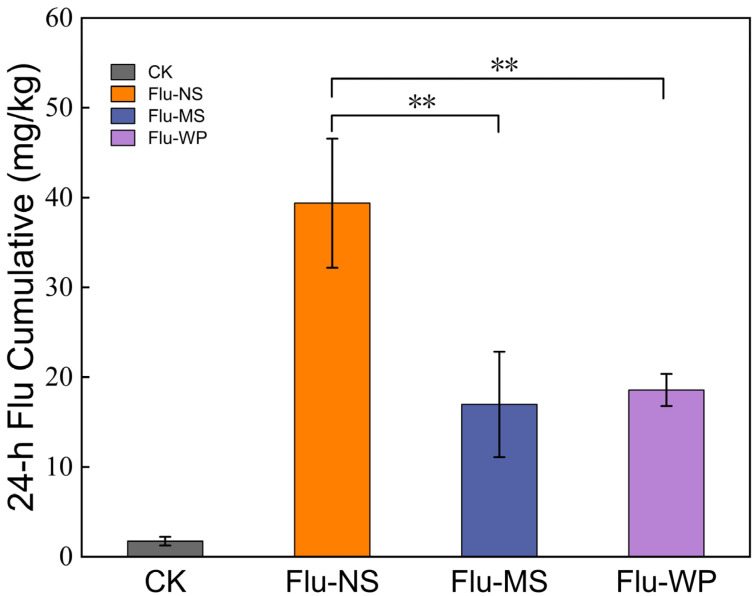
FLU uptake in *B. cinerea*. Different-size FLU were tested at 0.4 μg/mL. FLU accumulation was measured after 24 h incubation. Asterisks indicate FLU-NS showing a significantly different FLU accumulation of *p* < 0.05 from the other treatments. Error bars represent the standard deviation in the biological triplicates for each condition.

To gain insight into the mechanisms underlying the differences in the antifungal activity of formulations with varying particle sizes, fluopyram uptake tests were employed to measure the intracellular accumulation of these formulations. The cells in each test were starved via exposure to a glucose-depleted medium prior to FLU incubation, thus exhausting the cellular ATP. The incubation of FLU was then conducted under glucose-depleted conditions, to maximize the pesticide uptake, with the elimination of the efflux behavior of *B. cinerea*. When compared to the FLU-MS and FLU-WP group, FLU-NS extremely significantly improved the mycelial-ball uptake of FLU at 24 h (*p* < 0.01) (Figure 8). 

The 24-h treatment assay involving mycelial balls being treated with FLU-NS demonstrated the highest uptake of FLU, while those treated with FLU-MS and FLU-WP displayed similar uptake amounts, which were lower than that of FLU-NS. The formulations with nanoparticles notably increased the amount ingested by the pathogenic fungi. This indicates that the particle size of fluopyram has a significant effect on its uptake and accumulation in fungal cells. Specifically, formulations with particle sizes at the nanoscale are more likely to be ingested than those at the microscale, which is consistent with the differences in the antifungal activity of FLU formulations with different particle sizes against *B. cinerea* and *A. solani.*

These observations indicate that the pesticide was more likely to be ingested by pathogenic fungi as the particle size was reduced from 10 μm to 300 nm; thus, the pesticide activity and cell uptake effect could be markedly improved. In contrast, the antifungal activity and cell uptake of the pesticide hardly improved when the pesticide particle size was reduced from 10 μm to 3 μm. Some related studies found that the efficiency of the cellular uptake strongly depended on the particle size. The smaller the mesoporous silica nanoparticle (MSN) size, the more easily was absorbed and transmitted by plants [36]. Therefore, the nanosizing of pesticides appears to be a viable strategy to enhance efficiency without increasing the amount of pesticide used.

This research indicates that FLU-NS has a significantly higher likelihood of penetrating the pathogen interior in comparison to FLU-MS and FLU-WP. It is imperative to discuss the pathway and mechanism via which FLU-NS bypasses the fungal cell wall and plasma membrane and is transported to the inner membrane of the mitochondria. Previous studies show that drug particles are primarily taken up by cells through passive diffusion [37], active transport [38], or facilitated diffusion [39,40]. Therefore, it is vital to determine the mechanism via which FLU-NS enters the cell, whether it is through passive diffusion, active transport, or facilitated diffusion. Understanding the mechanism of FLU-NS entering the cell could have major implications in the development of more effective treatments for fungal infections.

## 3. Materials and Methods

### 3.1. Materials

Fluopyram (97.5% purity) was purchased from Bayer (China) Co., Ltd. (Leverkusen, Germany). 1-Dodecanesulfonic acid sodium salt (SDS), sodium dodecyl benzene sulfonate (SDBS), sodium lignosulfonate (SL, methoxy group: 5~7%, carbon: 45~47%), hydroxypropyl methyl cellulose (HPMC, hydroxylpropoxyl: 4~12%, methoxy: 19~24%, 100 mPa.s), sodium salt of polynaphthalene sulphonic acid (NNO), white carbon, diatomite, glucose, sucrose, mannitol, anhydrous d-trehalose, and sorbitol were obtained from Shanghai Macklin Biochemical Co., Ltd. (Shanghai, China). Pluronic F127 was obtained from Sigma-Aldrich (Shanghai, China). Polycarboxylate (TERSPERSE 2700) was provided by Huntsman Technology Corporation (USA). Morwet D425 and Morwet EFW were provided by Nanjing Jierun Technology Co., Ltd. (Nanjing, China). LC/MS-grade and HPLC-grade methanol and acetonitrile were obtained from Thermo Fisher Scientific (Waltham, MA, USA). Ultrapure water was used in all experiments. Qiagen RLT Lysis solution was purchased from Beijing Huaxia Ocean Technology Co., Ltd. (Beijing, China).

### 3.2. Preparation of Fluopyram Solid Formulation

A fluopyram nanosolid formulation was developed through the integration of wet medium milling and freeze drying. Specifically, the procedure involved mixing fluopyram powder with a specific surfactant (selected from SL, D425, SDS, SDBS, F127, HPMC, or 2700) in an aqueous solution. The resulting mixture was then poured into a grinding tank. The mixture was then subjected to high-energy planetary ball milling (FP2000E, FOCUCY, Hunan, China) at 500 rpm for 30 min. The suspension was processed in a nanomilling apparatus (Tyee, TBM-0.3, Shanghai, China) at 2500 rpm. The suspension containing the cryoprotectant was lyophilized for 24 h, to produce a nanosolid formulation.

A fluopyram microsolid formulation was prepared through the dispersing of the fluopyram powder in an aqueous HPMC solution, followed by grinding in the high-energy planetary ball mill (FOCUCY) at 300 rpm for 30 min. The suspension containing the cryoprotectant was then transferred to a lyophilizer and freeze dried for 24 h, resulting in a microsolid formulation.

To obtain the fluopyram wettable powder (WP), a high-speed universal pulverizer and a jet grinding mill (SJM-509, KS-Unique, Kunshan, China) were employed. The pulverizer was loaded with the fluopyram, carrier, dispersant, surfactant, and stabilizer in the prescribed amounts (fluopyram 20%, NNO 3%, EFW 3%, carrier white carbon 10%, and diatomite supplementation to 100%), and the mixture was then crushed for 1 min. Subsequently, the mixture was superfinely pulverized using the jet grinding mill (KS-Unique), with an airflow of 0.25 m^3^/min and a grinding pressure of 0.3 MPa.

### 3.3. Particle Size and ζ Potential

The FLU solid formulations were assessed for their particle size, polydispersity index (PDI), and potential, using a Malvern Zetasizer Nano^®^ ZS90 autosampler (Malvern Instruments, Malvern, UK). The samples were balanced in the instrument for 120 s at ambient temperature. The data were analyzed using Dispersion Technology Software Version 8.02 (Malvern Instruments, Malvern, UK).

### 3.4. Morphological Characterization via SEM

Using a scanning electron microscope (SEM; SU8020, Hitachi, Tokyo, Japan) with an acceleration voltage of 5 kV, the morphology of the FLU solid formulations was investigated. A clean silicon wafer was used as the substrate for the sample, which was then dried at room temperature and coated with gold using a sputter coater (EM ACE600, Leica, Weztlar, Germany).

### 3.5. Powder X-ray Diffraction Analysis

With the use of a diffractometer with a Cu Kα radiation source (D8 ADVANCE, Bruker AXS Inc., Karlsruhe, Germany), the crystalline states of the samples were examined. With the use of a detection rate of 0.2 °/min and a 2θ range of 5 to 50 °, the instrument was operated at 40 kV and 40 mA current.

### 3.6. Antifungal Activity Assays

The antifungal activities of *B. cinerea* and *A. solani* (provided by the College of Plant Protection, China Agricultural University, Beijing, China) were evaluated with the use of a PDA assay. Fluopyram solid formulations were prepared at the following concentrations: 0.50, 2.50, 10.0, 25.0, and 50.0 μg/mL for *B. cinerea*; and 0.031, 0.125, 0.50, 2.00, and 8.00 μg/mL for *A. solani*. A mycelial disc with a diameter of 5 mm was cultured on the test medium for 5 days at 25 ± 1 °C. The crisscross approach was used to gauge the mycelial growth’s diameter. With the use of SPSS v26 statistical software (IBM Corp., Armonk, NY, USA), the toxicity regression equations and the concentration for 50% of the maximal effect (EC50) were computed via probit analysis. All experiments were repeated in triplicate.

### 3.7. Different-Size-FLU Uptake by B. cinerea

Uptake experiments were conducted using the filamentous fungus *B. cinerea* to evaluate the intracellular accumulation of fluopyram of various sizes.

*B. cinerea* cultures were propagated using PDA plates at a temperature of 25 °C. To obtain conidia, 0.01% Tween 20 was added to sporulating plates of 7-day-old agar plate cultures. Afterward, the conidia were added to 50-mL conical tubes containing 5 mL of YAD medium with 2% glucose. The tubes were then shaken at 180 rpm for 48 h, which led to the formation of fungal balls that were around 3 mm in diameter, comprised of filamentous mycelial masses. The clusters of mycelium were moved to microcentrifuge tubes of 2 mL in volume and cleaned via undergoing centrifugation three times. Thereafter, the fungal ball pellets underwent a 2 h glucose-deprivation phase in a fresh YNB complete medium without glucose to de-energize the cells.

After a glucose-deprivation period, the pesticide treatment mixes were composed of 10 mL of YNB, fungal balls, and 20 μL of diluted FLU (200 μg/mL), resulting in a final FLU concentration of 0.4 μg/mL. The EC_50_ of FLU against cinerea was greater than 5 µg/mL, suggesting that the fluopyram concentration used for the uptake assay would not affect the cell viability.

After 24 h of incubation with FLU, the samples were vacuum-filtered through pre-weighed and wetted filter paper, followed by a rinse with 10 mL of ultrapure water to remove the FLU from the surfaces of the cells. The filtered fungal balls were then dried in an oven at 70 °C for 24 h. The dry mass of each fungal sample was determined via the re-weighing of the dried filtered products, which were transferred to a 50 mL centrifuge tube with 8 mL of acetonitrile and 2 mL of RLT lysis solution, sonicated for 30 min, and shaken at 180 rpm for 2 h, followed by centrifugation at 12,000 rpm for 10 min. Subsequently, the supernatant was filtered through a 0.22-μm organic membrane, to obtain samples for HPLC-MS/MS analysis.

The uptake concentration of FLU was measured via the determination of the accumulation per unit dry weight of the sample (mg/kg of mycelial mass). 

### 3.8. HPLC–MS/MS Conditions

A 1200/6410B HPLC-MS/MS (Agilent, Santa Clara, CA, USA) equipped with an Athena C18-WP column (CNW, 2.1 mm × 50 mm id, 3 μm) was used to determine the concentrations of fluopyram. Acetonitrile and 0.1% formic acid water were used as the mobile phases. Chromatographic separation was performed using a mobile phase consisting of acetonitrile and water with 0.1% formic acid (80:20, *v*/*v*) at a flow rate of 0.3 mL/min. The injection volume was 5 µL, and the column temperature was 30 °C.

Mass spectrometry (MS) was performed with the use of a 6410 B Triple Quadrupole mass spectrometer (Agilent, Santa Clara, CA, USA). ESI was used to run the instrument in positive-ion mode. Mass Hunter software (Agilent, version B.03.01) was used to control the instrument settings, data acquisition, and processing. The source parameters were optimized to a dry temperature of 350 °C, drying gas flow rate of 10.0 L/min, and capillary voltage of 4 kV. Two multi-response monitoring (MRM) ion transitions (*m*/*z* 397.1→173.1, *m*/*z* 397.1→208.2) were selected for confirmation, and the more-sensitive ion transition (*m*/*z* 397.1→173.1) was used for quantification.

### 3.9. Statistical Analysis

An unpaired two-tailed Student’s *t*-test was used to evaluate disparities between the groups of samples, and a *p*-value below 0.05 was deemed a statistically significant difference.

### 3.10. Thermal Storage Stability

The stability of three FLU formulations was tested according to the national standard of the People’s Republic of China GB/T 19136-2021 “Testing method of the accelerated storage stability at elevated temperature for pesticides”.

## 4. Conclusions

In this study, three solid formulations (FLU-NS, FLU-MS, and FLU-WP) of fluopyram with different particle sizes were prepared via combining wet media milling, freeze drying, and jet milling. The efficacy of these three formulations in mediating the antifungal activity against *B. cinerea* (Gray mold) and *A. solani* was assessed. Our study demonstrated that nanopesticides are more effective than conventional (coarser) pesticides because of their enhanced ability to cross the cell walls of pathogenic fungi and reach their intracellular targets. This improved efficacy has the potential to revolutionize the development and application of pesticide formulations, specifically those with intracellular targets.

## Figures and Tables

**Table 1:** The effects of different surfactants on the mean size and PDI of the suspension.

Surfactant	Mean Size (nm)	PDI
SL	602 ± 27.9	0.343 ± 0.047
F127	575 ± 34.3	0.159 ± 0.056
D425	580 ± 19.4	0.121 ± 0.023
SDS	831 ± 27.3	0.473 ± 0.069
SDBS	779 ± 27.3	0.207 ± 0.053
2700	623 ± 3.7	0.208 ± 0.039
HPMC	1074 ± 42.5	0.647 ± 0.099

Note: Data in the table are mean ± standard deviation.

**Table 2:** The effect of the different ratios of surfactant to fluopyram on the mean size of the suspension.

AI:Surfactant	Mean Size (nm)	PDI
2:1	759 ± 46.70	0.502 ± 0.158
5:1	732 ± 12.17	0.376 ± 0.013
7.5:1	576 ± 27.08	0.307 ± 0.021
10:1	580 ± 19.40	0.121 ± 0.023
15:1	719 ± 73.37	0.351 ± 0.018
20:1	---	---

Note: Data in the table are mean ± standard deviation; --- indicates agglomeration or precipitation in the suspension.

**Table 3:** Effects of different rotating speeds on the mean size of the suspension.

Parameter	Rotating Speed (rpm)		
	300	400	500
Mean size (nm)	2991 ± 171.2	1897 ± 125.7	1796 ± 129.5
PDI	0.268 ± 0.143	0.129 ± 0.057	0.087 ± 0.065

**Table 4:** The antifungal activity of three fluopyram solid formulations against *Botrytis cinerea* and *Alternaria solani*.

Fungal Strain	Formulations	Regressive Equation	R^2^	EC_50_ (μg/mL)	95% Confidence Interval	Toxicity Index
*Botrytis* *cinerea*	FLU-NS	Y = −0.397 + 0.543X	0.959	5.389	2.861~9.459	1.97
	FLU-MS	Y = −0.751 + 0.753X	0.970	9.939	6.853~15.496	1.07
	FLU-WP	Y = −0.770 + 0.750X	0.967	10.637	7.043~16.734	1.00
*Alternaria* *solani*	FLU-NS	Y = 0.383 *+* 0.625X	0.933	0.244	0.120~0.439	3.42
	FLU-MS	Y = 0.212 *+* 0.985X	0.984	0.609	0.407~0.921	1.37
	FLU-WP	Y = 0.064 *+* 0.813X	0.948	0.835	0.524~1.405	1.00

## Data Availability

Not applicable.

## Sample Availability

Samples of the compounds are available from the authors.

## References

[1] Kumar R., Kumar N., Rajput V.D., Mandzhieva S., Minkina T., Saharan B.S., Kumar D., Sadh P.K., Duhan J.S. (2022). Advances in Biopolymeric Nanopesticides: A New Eco-Friendly/Eco-Protective Perspective in Precision Agriculture. Nanomaterials.

[2] Tong Y., Shao L., Li X., Lu J., Sun H., Xiang S., Zhang Z., Wu Y., Wu X. (2018). Adhesive and Stimulus-Responsive Polydopamine-Coated Graphene Oxide System for Pesticide-Loss Control. J. Agric. Food Chem..

[3] Parisi C., Vigani M., Rodríguez-Cerezo E. (2015). Agricultural Nanotechnologies: What are the current possibilities?. Nano Today.

[4] Chen M., Zhou S., Zhu Y., Sun Y., Zeng G., Yang C., Xu P., Yan M., Liu Z., Zhang W. (2018). Toxicity of carbon nanomaterials to plants, animals and microbes: Recent progress from 2015-present. Chemosphere.

[5] Chen H., Yada R. (2011). Nanotechnologies in agriculture: New tools for sustainable development. Trends Food Sci Tech..

[6] Nuruzzaman M., Rahman M.M., Liu Y., Naidu R. (2016). Nanoencapsulation, nano-guard for pesticides: A new window for safe application. J. Agric. Food Chem..

[7] Zhao X., Cui H., Wang Y., Sun C., Cui B., Zeng Z. (2018). Development Strategies and Prospects of Nano-based Smart Pesticide Formulation. J. Agric. Food Chem..

[8] Merisko-Liversidge E., Liversidge G.G. (2011). Nanosizing for oral and parenteral drug delivery: A perspective on formulating poorly-water soluble compounds using wet media milling technology. Adv. Drug Deliv. Rev..

[9] Vardaka E., Ouranidis A., Nikolakakis I., Kachrimanis K. (2021). Development of agomelatine nanocomposite formulations by wet media milling. Eur. J. Pharm. Sci..

[10] Toziopoulou F., Malamatari M., Nikolakakis I., Kachrimanis K. (2017). Production of aprepitant nanocrystals by wet media milling and subsequent solidification. Int. J. Pharm..

[11] Wu L., Zhang J., Watanabe W. (2011). Physical and chemical stability of drug nanoparticles. Adv. Drug Deliv. Rev..

[12] Skoglund S., Lowe T.A., Hedberg J., Blomberg E., Wallinder I.O., Wold S., Lundin M. (2013). Effect of Laundry Surfactants on Surface Charge and Colloidal Stability of Silver Nanoparticles. Langmuir.

[13] Zhong X., Duan F. (2015). Surfactant-Adsorption-Induced Initial Depinning Behavior in Evaporating Water and Nanofluid Sessile Droplets. Langmuir.

[14] Singh H., Aswal V.K. (2021). Tuning of micelle adsorption on nanoparticles by combination of surfactants. J. Appl. Phys..

[15] Tian C., Feng J., Prud’homme R.K. (2020). Adsorption dynamics of polymeric nanoparticles at an air-water interface with addition of surfactants. J. Colloid. Interface Sci..

[16] Li X., Qin Y., Liu C., Jiang S., Xiong L., Sun Q. (2016). Size-controlled starch nanoparticles prepared by self-assembly with different green surfactant: The effect of electrostatic repulsion or steric hindrance. Food Chem..

[17] Li B., Ju M., Dou X., Li N., Zhang W., Sun Z., Yu K., Wang J., Wang Z. (2022). Assessing nanoparticle-surfactant-salt synergistic effects on droplet–droplet electrocoalescence by molecular dynamics simulations. J. Mol. Liq..

[18] Dean R., Van Kan J.A., Pretorius Z.A., Hammond-Kosack K.E., Di Pietro A., Spanu P.D., Rudd J.J., Dickman M., Kahmann R., Ellis J. (2012). The Top 10 fungal pathogens in molecular plant pathology. Mol. Plant Pathol..

[19] Acero F.J., Carbu M., El-Akhal M.R., Garrido C., Gonzalez-Rodriguez V.E., Cantoral J.M. (2011). Development of proteomics-based fungicides: New strategies for environmentally friendly control of fungal plant diseases. Int. J. Mol. Sci..

[20] Kozhar O., Peever T.L. (2018). How Does Botrytis cinerea Infect Red Raspberry?. Phytopathology.

[21] South K.A., Peduto Hand F., Jones M.L. (2020). Beneficial Bacteria Identified for the Control of Botrytis cinerea in Petunia Greenhouse Production. Plant Dis..

[22] Horsefield R., Yankovskaya V., Sexton G., Whittingham W., Shiomi K., Omura S., Byrne B., Cecchini G., Iwata S. (2006). Structural and computational analysis of the quinone-binding site of complex II (succinate-ubiquinone oxidoreductase): A mechanism of electron transfer and proton conduction during ubiquinone reduction. J. Biol. Chem..

[23] Cecchini G. (2003). Function and structure of complex II of the respiratory chain. Annu. Rev. Biochem..

[24] Matsson M., Hederstedt L. (2001). The Carboxin-Binding Site on Paracoccus denitrificans Succinate:Quinone Reductase Identified by Mutations. J. Bioenerg. Biomembr..

[25] Villa F., Cappitelli F., Cortesi P., Kunova A. (2017). Fungal Biofilms: Targets for the Development of Novel Strategies in Plant Disease Management. Front. Microbiol..

[26] Torrano A.A., Herrmann R., Strobel C., Rennhak M., Engelke H., Reller A., Hilger I., Wixforth A., Bräuchle C., Torrano A.A. (2016). Cell membrane penetration and mitochondrial targeting by platinum-decorated ceria nanoparticles. Nanoscale.

[27] Cuculis L., Meredyth N.A., Frey S.L. (2012). Nanoparticle and Surfactant Interactions with Model Cell Membranes. Biophys. J..

[28] Voci S., Gagliardi A., Salvatici M.C., Fresta M., Cosco D. (2022). Influence of the Dispersion Medium and Cryoprotectants on the Physico-Chemical Features of Gliadin- and Zein-Based Nanoparticles. Pharmaceutics.

[29] Amis T.M., Renukuntla J., Bolla P.K., Clark B.A. (2020). Selection of Cryoprotectant in Lyophilization of Progesterone-Loaded Stearic Acid Solid Lipid Nanoparticles. Pharmaceutics.

[30] Umerska A., Paluch K.J., Santos-Martinez M.J., Corrigan O.I., Medina C., Tajber L. (2018). Freeze drying of polyelectrolyte complex nanoparticles: Effect of nanoparticle composition and cryoprotectant selection. Int. J. Pharm..

[31] Wang L., Ma Y., Gu Y., Liu Y., Zhao J., Yan B., Wang Y. (2018). Cryoprotectant choice and analyses of freeze-drying drug suspension of nanoparticles with functional stabilisers. J. Microencapsul..

[32] Fan F., Roos Y.H. (2016). Crystallization and structural relaxation times in structural strength analysis of amorphous sugar/whey protein systems. Food Hydrocoll..

[33] Ito A., Konnerth C., Schmidt J., Peukert W. (2016). Effect of polymer species and concentration on the production of mefenamic acid nanoparticles by media milling. Eur. J. Pharm. Biopharm..

[34] George M., Ghosh I. (2013). Identifying the correlation between drug/stabilizer properties and critical quality attributes (CQAs) of nanosuspension formulation prepared by wet media milling technology. Eur. J. Pharm. Sci..

[35] Dong Y., Zhang D., Li D., Jia H., Qin W. (2023). Control of Ostwald ripening. Sci. China Mater..

[36] Xu Y., Xu C., Huang Q., Cao L., Teng F., Zhao P., Jia M. (2021). Size Effect of Mesoporous Silica Nanoparticles on Pesticide Loading, Release, and Delivery in Cucumber Plants. Appl. Sci..

[37] Linnane E., Haddad S., Melle F., Mei Z., Fairen-Jimenez D., Linnane E. (2022). The uptake of metal–organic frameworks: A journey into the cell. Chem. Soc. Rev..

[38] Jia J.-L., Jin X.-Y., Zhu L., Zhang Z.-X., Liang W.-L., Wang G.-D., Zheng F., Wu X.-Z., Xu H.-H. (2017). Enhanced intracellular uptake in vitro by glucose-functionalized nanopesticides. N. J. Chem..

[39] Esquivel B.D., White T.C. (2017). Accumulation of azole drugs in the fungal plant pathogen *Magnaporthe oryzae* is the result of facilitated diffusion influx. Front. Microbiol..

[40] Esquivel B.D., Smith A.R., Zavrel M., White T.C. (2015). Azole drug import into the pathogenic fungus *Aspergillus fumigatus*. Antimicrob. Agents Chemother..

